# Meta-Analysis of the Associations of p-Cresyl Sulfate (PCS) and Indoxyl Sulfate (IS) with Cardiovascular Events and All-Cause Mortality in Patients with Chronic Renal Failure

**DOI:** 10.1371/journal.pone.0132589

**Published:** 2015-07-14

**Authors:** Cheng-Jui Lin, Vincent Wu, Pei-Chen Wu, Chih-Jen Wu

**Affiliations:** 1 Division of Nephrology, Department of Internal Medicine, Mackay Memorial Hospital, Taipei, Taiwan; 2 Mackay Junior College of Medicine, Nursing and Management, Taipei, Taiwan; 3 Division of Nephrology, Department of Internal Medicine, National Taiwan University Hospital, Taipei, Taiwan; 4 Graduate Institute of Medical Science, Taipei Medical University, Taipei, Taiwan; 5 Department of Medicine, Mackay Medical College, New Taipei City, Taiwan; Hospital Universitario de La Princesa, SPAIN

## Abstract

**Background:**

Indoxyl sulfate (IS) and p-cresyl sulfate (PCS) are protein-bound uremic toxins that increase in the sera of patients with chronic kidney disease (CKD), and are not effectively removed by dialysis. The purpose of this meta-analysis was to investigate the relationships of PCS and IS with cardiovascular events and all-cause mortality in patients with CKD stage 3 and above.

**Methodology/Principle Findings:**

Medline, Cochrane, and EMBASE databases were searched until January 1, 2014 with combinations of the following keywords: chronic renal failure, end-stage kidney disease, uremic toxin, uremic retention, indoxyl sulfate, p-cresyl sulfate. Inclusion criteria were: 1) Patients with stage 1 to 5 CKD; 2) Prospective study; 3) Randomized controlled trial; 4) English language publication. The associations between serum levels of PCS and IS and the risks of all-cause mortality and cardiovascular events were the primary outcome measures. Of 155 articles initially identified, 10 prospective and one cross-sectional study with a total 1,572 patients were included. Free PCS was significantly associated with all-cause mortality among patients with chronic renal failure (pooled OR = 1.16, 95% CI = 1.03 to 1.30, *P* = 0.013). An elevated free IS level was also significantly associated with increased risk of all-cause mortality (pooled OR = 1.10, 95% CI = 1.03 to 1.17, *P* = 0.003). An elevated free PCS level was significantly associated with an increased risk of cardiovascular events among patients with chronic renal failure (pooled OR = 1.28, 95% CI = 1.10 to 1.50, *P* = 0.002), while free IS was not significantly associated with risk of cardiovascular events (pooled OR = 1.05, 95% CI = 0.98 to 1.13, *P* = 0.196).

**Conclusions/Significance:**

Elevated levels of PCS and IS are associated with increased mortality in patients with CKD, while PCS, but not IS, is associated with an increased risk of cardiovascular events.

## Introduction

The prevalence of cardiovascular disease (CVD) is markedly higher in patients with chronic kidney disease (CKD) [1], and CVD disease is the primary cause of death in CKD patients, especially in those with end stage renal disease (ESRD) [2,3]. Traditional CVD risk factors such as hypertension and diabetes and non-traditional such as hyperhomocysteinemia, however, do not fully account for the increased CVD risk in patients with CKD [4,5].

Impairment of renal function results in the retention of a large number of compounds which are normally excreted in the urine [6,7]. While some compounds such as urea are removed by dialysis in patients with ESRD, many others are not [6,7]. These retained compounds are called uremic retention solutes or uremic toxins [6,7].

Indoxyl sulfate (IS) is a protein-bound uremic solute resulting from bacterial metabolism of dietary tryptophan to indole [6,8,9]. It is normally cleared by renal proximal tubular secretion; but in patients with CKD impaired renal function can lead to its accumulation [6,8,9]. Another uremic solute, p-cresyl sulfate (PCS) is synthesized by intestinal anaerobic bacteria from the amino acids tyrosine and phenylalanine, and like IS it is normally secreted in the urine [6,8,9]. Both PCS and IS cannot be removed efficiently by hemodialysis or peritoneal dialysis because protein binding limits their clearance [10]. Levels of IS and PCS increase with the severity of CKD, and both have been shown to have a strong negative correlation with renal function (estimated glomerular filtration rate [eGFR]) in patients with CKD [11–14]. Furthermore, studies have shown that IS and PCS are associated with CVD, mortality, and deterioration of renal function in patients with CKD [15–18].

The purpose of this systematic review of the literature and meta-analysis is to investigate the relationships of PCS and IS levels with cardiovascular events and all-cause mortality in patients with CKD.

## Materials and Methods

### Search strategy and selection criteria

**Fig 1:**
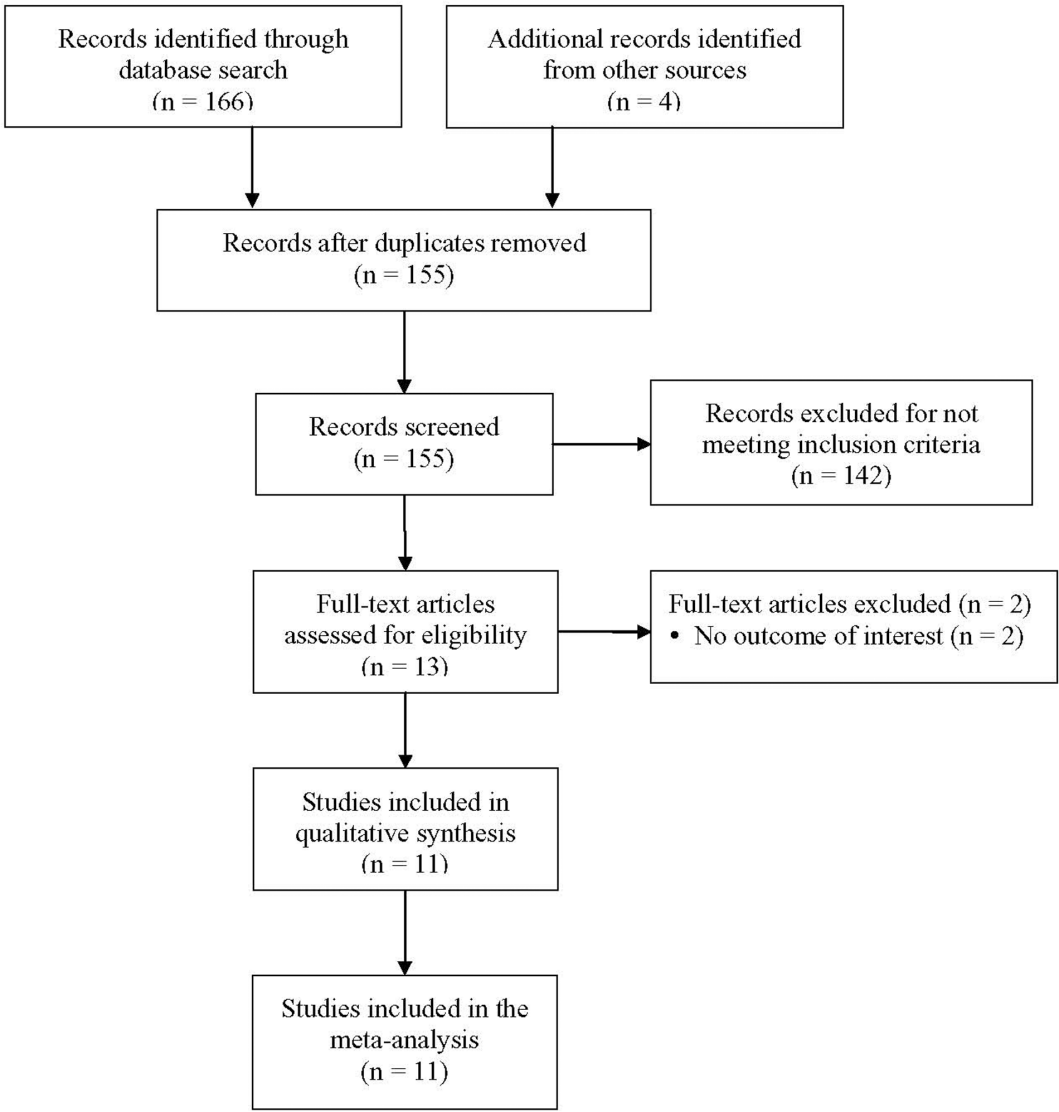
Flow chart of study selection.

Medline, Cochrane, and EMBASE databases were searched until January 1, 2014 with combinations of the following keywords: chronic renal failure, end-stage kidney disease, uremic toxin, uremic retention, indoxyl sulfate, p-cresyl sulfate. Reference lists of relevant published articles (as “other resources” in Fig 1) were also searched. Inclusion criteria were: 1) patients with stage 1 to 5 CKD, or undergoing hemodialysis or peritoneal dialysis; 2) prospective study; 3) randomized controlled trial; 4) English language publication. Case reports, comments, editorials, letters, and non-English publications were excluded.

### Study selection and data extraction

Studies were identified via the search strategy by two independent reviewers. When there was uncertainty regarding eligibility, a third reviewer was consulted. The following data were extracted from studies that met the inclusion criteria: the name of the first author, year of publication, study design, number of participants in each group, participants’ age, gender, and CKD stage, and IS, PCS, albumin, creatinine, and parathyroid hormone (PTH) levels.

### Quantitative data synthesis and outcome measures

The associations between serum levels of PCS and IS, and the risks of all-cause mortality and cardiovascular events were the primary outcome measures. The risks of all-cause mortality and cardiovascular events associated with albumin, creatinine, and PTH levels were also determined for comparison. The odds ratio (OR) and/or hazard ratio (HR) and corresponding 95% confidence interval (CI) from each study were used for evaluation. For prospective studies, the HR was considered as the OR in the statistical analysis since there was barely numeric difference between the two [19]. Heterogeneity among studies was assessed by calculating Cochran Q and the I^2^ statistic. For the Q statistic, *P* < 0.10 was considered to indicate statistically significant heterogeneity. The I^2^ statistic indicates the percentage of the observed between-study variability caused by heterogeneity. Heterogeneity determined using the I^2^ statistic was defined as follows: 0 to 24% = no heterogeneity; 25 to 49% = moderate heterogeneity; 50 to 74% = large heterogeneity; and 75 to 100% = extreme heterogeneity. If heterogeneity existed between studies (a Q statistic with *P* < 0.1 [20] or an I^2^ statistic > 50% [21]), a random-effects model (DerSimonian-Laird method) was performed [22]. Otherwise, a fixed-effect model was used (Mantel-Haenszel method). Combined ORs were calculated, and a two-sided *P* value < 0.05 was considered to indicate statistical significance.

The associations between serum levels of free PCS and IS and the risk of all-cause mortality, as well as cardiovascular events, were further examined by sensitivity analysis which was performed based on the leave-one-out approach. Publication bias was assessed by constructing funnel plots for the associations between the serum levels of free PCS and IS, and the risk of all-cause mortality and cardiovascular events. It was also quantitatively detected by Egger’s test [23]. The absence of publication bias is indicated by the data points forming a symmetric funnel-shaped distribution and *P* > 0.10 in Egger’s test. Moreover, the Duval and Tweedie’s trim-and-fill method was used to adjust for potential publication bias. The trim-and-fill method formalizes the interpretation of any asymmetry in the funnel plot by imputing suspected missing studies and calculating an adjusted result. The adjusted result is neither intended to find the values of missing studies, nor to give a better effect size estimate in itself, but is used as a form of sensitivity analysis to help ascertain the probable effect of publication bias on the meta-analysis [24–26]. All statistic analyses were performed using the Comprehensive Meta-Analysis statistical software, version 2.0 (Biostat, Englewood, NJ, USA).

## Results

### Literature search

A summary of the literature search and study selection is shown in Fig 1. After initially identifying 155 articles after the removal of duplicates, 142 were excluded as they did not meet the stringent inclusion criteria. Thirteen articles were thus included in the full text review. Of the 13 articles, two were excluded as they did not provide data with respect to the outcome of interest [13,14]. Thus, eleven studies were included in the meta-analysis [11,12,15–18,27–31].

### Study characteristics

The basic characteristics of the 11 studies included in the meta-analysis are summarized in Table 1. Among the eleven studies included, there were 10 prospective studies and one cross-sectional study with a total of 1,572 patients. The total number of patients in each of the studies ranged from 46 to 521, and the follow-up duration of the prospective studies ranged from 20 months to 5 years. A summary of the serum levels of the five biomarkers (free PCS, free IS, albumin, creatinine, and PTH) is also presented in Table 1.

**Table 1:** Characteristics of studies included in the meta-analysis.

Author (Year)	Study Type	Number of Patients	Type of Patients	Age (y)	Male (%)	Follow-up Time	Free IS(mg/L)	Free PCS(mg/L)	Albumin (g/dL)	Creatinine (mg/dL)	Parathyroid Hormone (pg/mL)
Lin et al. (2013) [27]	Prospective	46	ESRD	47.4 ± 12.8	45.60%	5 y	4.3 ± 2.6	1.1 ± 1.0	3.9 ± 0.3	13.3 ± 3.4	382.9 ± 279.4
Lin et al. (2013) [28]	Prospective cohort	50	Hemodialysis	70.50 ± 3.45	NR	38 mo	4.27 ± 2.90	1.59 ± 1.12	4.05 ± 0.34	10.36 ± 2.32	291.44 ± 235.09
Melamed et al. (2013) [15]	Prospective cohort	521	Dialysis	58.3 ± 14.7	46.00%	NR	NR	NR	3.6 ± 0.4	7.3 ± 2.4	NR
Lin et al. (2012) [29]	Prospective cohort	70	Stable CKD (stages 3–5)	60.6 ± 9.7	48.60%	36 mo	NR	NR	4.02 ± 0.4	3.76 ± 2.7	132 ± 176
Wu et al. (2011) [16]	Prospective cohort	112	Hemodialysis	72.6 ± 6.3	69.60%	3.5 y	3.7 (0.01, 19.7) ^‡^	2.2 (0.01, 25.6) ^‡^	3.6 ± 0.4	NR	197 (10.1, 3104) ^‡^
Chen et al. (2012) [30]	Cross-sectional	91	Hemodialysis	57.6 ± 1.2	57.10%	NR	2.9 ± 0.2	2.1 ± 0.2	3.9 ± 0.3	11.3 ± 0.2	NR
Wu et al. (2011) [31]	Prospective observational	268	Pre-dialysis CKD	66.9 ± 12	42.50%	24 mo	NR	NR	3.9 ± 0.4	1.9 ± 1.4	89.9 (1, 692) ^‡^
Lin et al. (2010) [17]	Prospective	100	Hemodialysis	62.36 ± 8.03	42.00%	20 mo	4.42 ± 2.41	1.57 ± 0.95	4.05 ± 0.27	10.89 ± 2.07	335.53 ± 192.98
Liabeuf et al. (2010) [11]	Prospective cohort	139	Stage 1–5 CKD	67 ± 12	60%	779 ± 185 d	NR	2.6 (5.1)	3.8 (6)	NR	137 (138)
Barreto et al. (2009) [12]	Prospective cohort	139	Stage 1–5 CKD	67 ± 12	60%	605 ± 217 d	NR	2.6 (5.1)	3.8 (6)	NR	137 (138)
Bammens et al. (2006) [18]	Prospective	175	Stage 5 CKD	64.7 ± 1.1^†^	38.30%	30.1 mo	NR	2.59 ± 0.17	3.64 ± 0.03	8.6 ± 0.2	NR

Data are reported as mean ± standard deviation unless otherwise indicated.

ESRD, end stage renal disease; CKD, chronic kidney disease; IS, indoxyl sulfate; PCS, p-cresyl sulfate; NR, not reported.

^†^ Mean ± SEM.

^‡^ Median (range).

### Associations between biomarkers and all-cause mortality

**Fig 2:**
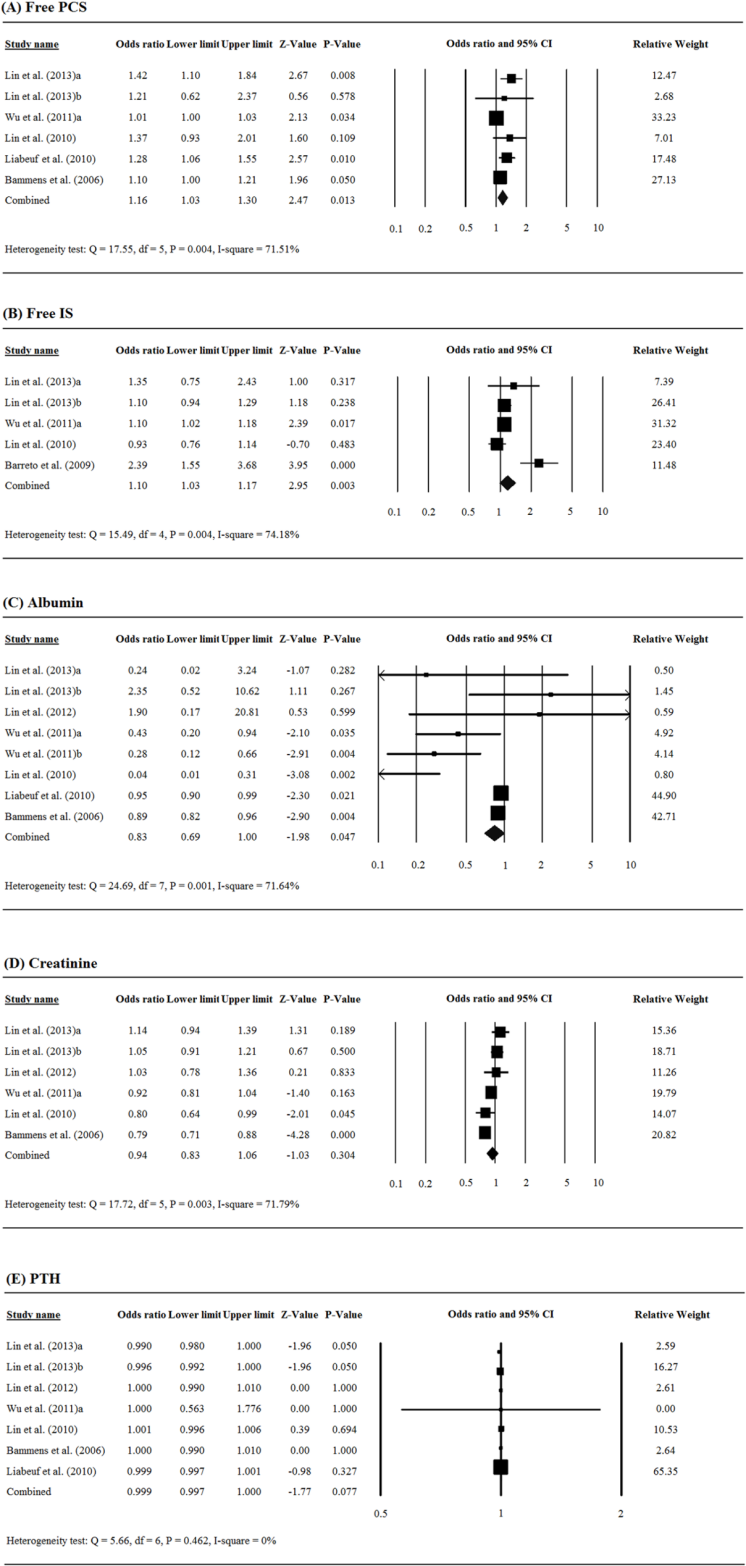
Forest plots of the associations between serum levels of renal biomarkers and the risk of all-cause mortality among patients with chronic renal failure: (A) free PCS, (B) free IS, (C) albumin, (D) creatinine, (E) PTH.

Free PCS was significantly associated with all-cause mortality among patients with chronic renal failure (pooled OR = 1.16, 95% CI = 1.03 to 1.30, *P* = 0.013, Fig 2A). An elevated free IS level was also significantly associated with increased risk of all-cause mortality (pooled OR = 1.10, 95% CI = 1.03 to 1.17, *P* = 0.003, Fig 2B). There was significant association between albumin level and all-cause mortality among patients with chronic renal failure (pooled OR = 0.83, 95% CI = 0.69 to 1.00, *P* = 0.047, Fig 2C), while there was no significant association between creatinine and PTH levels and all-cause mortality (creatinine: pooled OR = 0.94, 95% CI = 0.83 to 1.06, *P* = 0.304, Fig 2D; PTH: pooled OR = 0.999, 95% CI = 0.997 to 1.000, *P* = 0.077, Fig 2E).

### Associations between biomarkers and cardiovascular events

**Fig 3:**
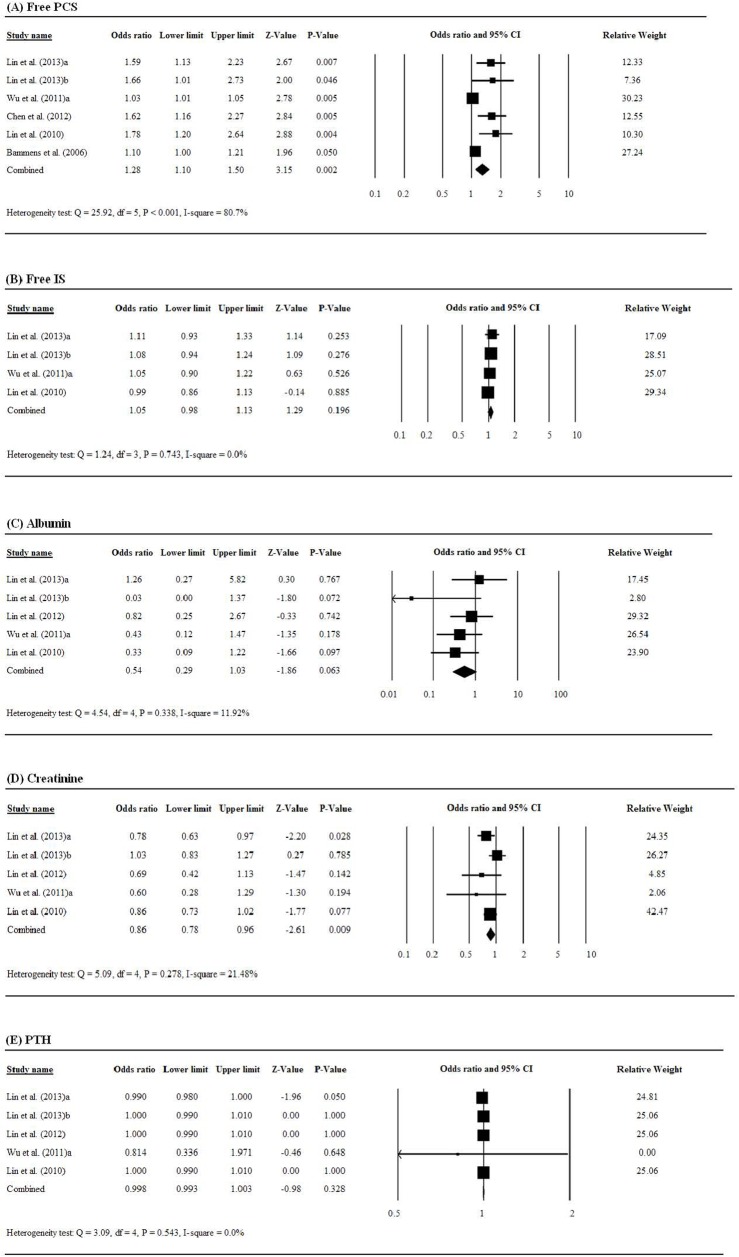
Forest plots of the associations between serum levels of renal biomarkers and the risk of cardiovascular events among patients with chronic renal failure: (A) free PCS, (B) free IS, (C) albumin, (D) creatinine, (E) PTH.

An elevated free PCS level was significantly associated with an increased risk of cardiovascular events among patients with chronic renal failure (pooled OR = 1.28, 95% CI = 1.10 to 1.50, *P* = 0.002, Fig 3A), while free IS was not significantly associated with risk of cardiovascular events (pooled OR = 1.05, 95% CI = 0.98 to 1.13, *P* = 0.196, Fig 3B). Of albumin, creatinine, and PTH, only creatinine level had a significantly negative association with the risk of cardiovascular events (pooled OR = 0.86, 95% CI = 0.78 to 0.96, *P* = 0.009, Fig 3C and 3D).

### Sensitivity analysis

**Fig 4:**
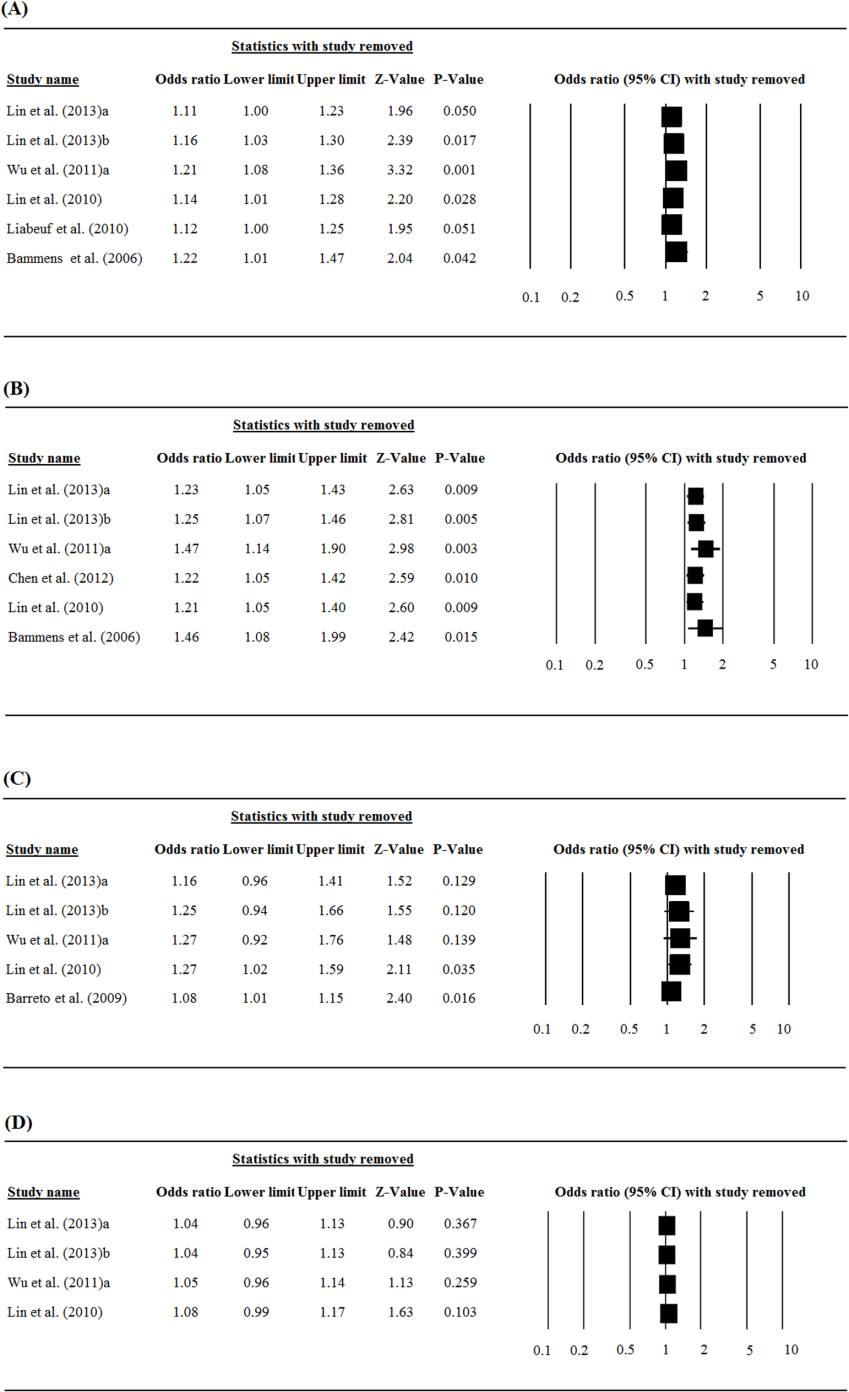
Sensitivity analysis performed by leave-one-out approach: (A) association between serum level of free PCS and the risk of mortality, (B) association between serum level of free PCS and the risk of cardiovascular events, (C) association between serum level of free IS and the risk of mortality, (D) association between serum level of free IS and the risk of cardiovascular events.

Results of the sensitivity analysis for the association between free PCS and IS levels and the risks of all-cause mortality and cardiovascular events in which the studies were omitted one-by-one, are shown in Fig 4. As a whole, the direction and magnitude of the pooled ORs did not vary markedly with removal of any study, indicating good reliability in this meta-analysis.

### Publication Bias

**Fig 5:**
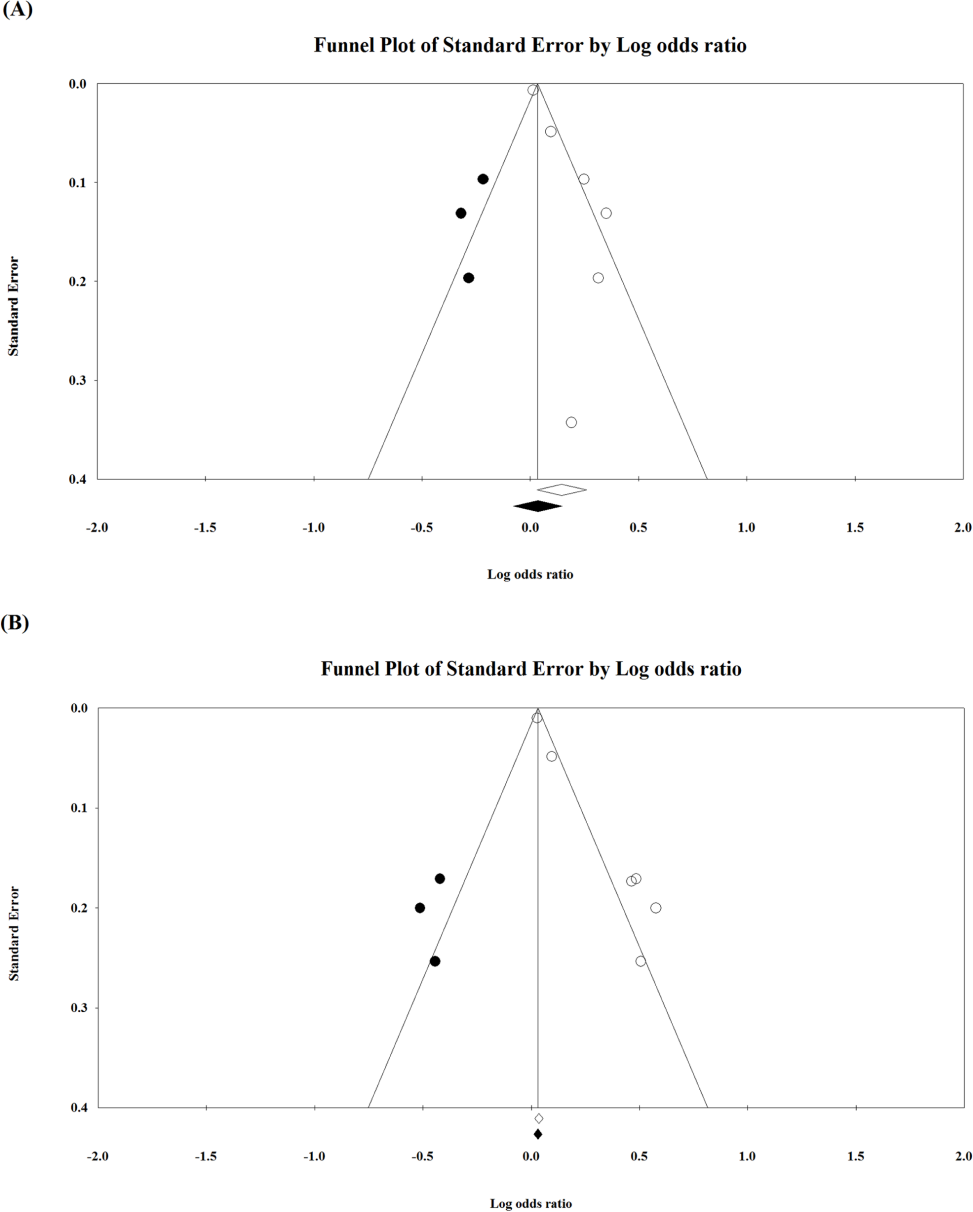
Funnel plot for evaluating publication bias regarding the association between free PCS and (A) mortality risk and (B) the risk of cardiovascular events. White circles represent observed studies, and black circles represent possibly missed studies imputed using Duval and Tweedie’s trim-and-fill method. White and black rhombuses represent observed and theoretical combined effect size, respectively.

The funnel plot for publication bias regarding the association between free PCS and mortality risk showed evidence of asymmetry and publication bias (Fig 5A). Egger’s test also indicated there was significant evidence of publication bias (t = 4.50, df = 4, *P* = 0.005). When the Duval and Tweedie’s trim-and-fill method was used to adjust for the effect of publication bias, and when possibly missed studies were imputed (Fig 5A), the adjusted point estimates of the OR decreased from 1.16 (95% CI = 1.03 to 1.30) to 1.03 (95% CI = 0.93 to 1.16). This suggests that publication bias may have exaggerated the observed effect size, and the significant association found between serum free PCS level and mortality risk should be interpreted with caution.

The funnel plot for publication bias regarding the association between free PCS and risk of cardiovascular events showed evidence of asymmetry and publication bias (Fig 5B). Egger’s test also indicated there was significant evidence of publication bias (t = 10.73, df = 4, *P* < 0.001). When the Duval and Tweedie’s trim-and-fill method was used to adjust for the effect of publication bias, and when possibly missed studies were imputed (Fig 5B), the adjusted point estimates of the OR decreased from 1.28 (95% CI = 1.10 to 1.50) to 1.10 (95% CI = 0.93 to 1.27). This suggests that publication bias may have exaggerated the observed effect size, and the significant association found between serum free PCS level and the risk of cardiovascular events should be interpreted with caution.

Due to the small number of studies (≤ 5), it was inappropriate to assess for publication bias using the funnel plot [25] with regard to the associations between free IS level and mortality risk or the risk of cardiovascular events.

## Discussion

PCS and IS are two of more than 80 uremic toxins that increase in the sera of patients with CKD and are not removed by dialysis [6]. Study has shown that the serum concentrations of IS and PCS in patients with CKD are 54 and 17 times higher, respectively, than in healthy individuals, and because they are bound to albumin only approximately 30% are eliminated by hemodialysis [7,10,32]. The results of this meta-analysis indicate that elevated levels of PCS and IS are associated with increased mortality in patients with CKD, while PCS, but not IS, is associated with an increased risk of cardiovascular events.

A growing number of publications have confirmed the negative effects of IS and PCS. Barretto et al. [12] studied 139 patients with stage 2 to 5D CKD and reported that IS level was inversely related to renal function, and high IS was significantly associated with cardiovascular and overall mortality after adjustment for age, gender, diabetes, albumin, hemoglobin, phosphate, and aortic calcification. In further analysis of the same 139 patients, Liabeuf et al. [11] found that higher free PCS levels were associated with mortality independent of factors such as age, vascular calcification, anemia, and inflammation. Meijer et al. [33] reported that PCS level was predictive of cardiovascular risk in 499 patients with mild to moderate CKD. A cross-section observational study by Rossi et al. [34] showed that more advanced stages of CKD were associated with progressive increases in total and free serum IS and PCS, and that their levels were independently associated with structural and functional markers of cardiovascular disease. In a prospective study of 200 patients with stage 1 to 5 CKD, Poesen et al. [35] found that urinary excretion of PCS was predictive of cardiovascular events independent of eGFR.

While most results of the studies included in this meta-analysis are generally consistent, there are some differences. Lin et al. [27] found that total PCS was associated with cardiovascular events, free PCS was associated with all-cause mortality, and total IS was associated with dialysis failure in stable peritoneal dialysis patients. In a separate study of elderly (> 65 years of age) patients receiving hemodialysis, Lin et al. [28] found that free and total PCS were significantly associated with cardiovascular events and the total PCS was associated with all-cause mortality. In a recent report (2014), Lin et al. [36] examined 72 pre-dialysis patients and found that a serum PCS level > 6 mg/L was associated with cardiovascular events and initiation of dialysis. Melamed et al. [15], however, found that elevated PCS levels (defined as greater than the population median) were not associated with either all-cause or cardiovascular mortality, and that elevated IS levels were associated with all-cause mortality, but not with cardiovascular mortality. Wu et al. [16] reported that free PCS was associated with all-cause and cardiovascular mortality after adjusting for traditional risk factors such as age, gender, and diabetes status in elderly patients receiving hemodialysis. Bammens et al. [18] also found that a higher level of free PCS was independently associated with mortality in hemodialysis patients.

While the number of publications supporting the predictive value of PCS and IS in patients with CKD is increasing, Vanholder et al. [37] have pointed out that the interpretation of the results may be affected by different cutoff values of PCS and IS, and low albumin concentrations in the study populations. To this end the authors performed a systematic review of the literature, and after excluding studies biased by albumin binding considerations they concluded that PCS and IS indeed play a role vascular and renal disease progression. The exact mechanisms by which elevated levels of IS and PCS contribute to CVD and mortality, however, have not been elucidated. Studies have suggested that IS and PCS may suppress the activity or activated leucocytes, inhibit the release of platelet-activating factor by macrophages, and contribute to endothelial dysfunction and oxidative stress [9,38,39].

There are limitations of this study that should be considered. Given the number of studies in the literature examining the roles of IS and PCS in outcomes of patients with CKD, the number of studies included in the meta-analysis is relatively small due to the relatively strict inclusion criteria. Publication bias may have exaggerated the significant association found between PCS level and the risk of cardiovascular events and all-cause mortality, but could not be assessed with respect to IS level and the risks of mortality and cardiovascular events due to the small number of studies. Furthermore, confounding factors (age, gender, diabetic or dialysis status etc.) of mortality for which multivariate analytic data were adjusted varied among studies, and additional multi-variable analyses for those factors with mortality association were not performed.

## Conclusions

This meta-analysis indicated that elevated levels of PCS and IS are associated with increased mortality in patients with CKD, and PCS, but not IS, is associated with an increased risk of cardiovascular events. Although it is clear that PCS and IS are associated with negative outcomes in patients with CKD, further studies are necessary to determine their exact roles and mechanisms, and possible treatment options to reduce their negative impact in patients with CKD.

## Supporting Information

S1 PRISMA Checklist (DOC)

## References

[1] Vanholder R, Massy Z, Argiles A, Spasovski G, Verbeke F, Lameire N; European Uremic Toxin Work Group. Chronic kidney disease as cause of cardiovascular morbidity and mortality. Nephrol Dial Transplant. 2005;20: 1048–1056. 15814534 10.1093/ndt/gfh813

[2] Weiner DE, Tighiouart H, Amin MG, Stark PC, MacLeod B, Griffith JL, et al Chronic kidney disease as a risk factor for cardiovascular disease and all-cause mortality: a pooled analysis of community-based studies. J Am Soc Nephrol. 2004;15: 1307–1315. 15100371 10.1097/01.asn.0000123691.46138.e2

[3] Levin A, Foley RN. Cardiovascular disease in chronic renal insufficiency. Am J Kidney Dis. 2000;36: S24–S30. 10.1053/ajkd.2000.19928 11118155

[4] Longenecker JC, Coresh J, Powe NR, Levey AS, Fink NE, Martin A, et al Traditional cardiovascular disease risk factors in dialysis patients compared with the general population: the CHOICE Study. J Am Soc Nephrol. 2002;13:1918–1927. 12089389 10.1097/01.asn.0000019641.41496.1e

[5] Clarke R, Daly L, Robinson K, Naughten E, Cahalane S, Fowler B, et al Hyperhomocysteinemia: an independent risk factor for vascular disease. N Engl J Med. 1991; 324: 1149–1155. 2011158 10.1056/NEJM199104253241701

[6] Duranton F, Cohen G, De Smet R, Rodriguez M, Jankowski J, Vanholder R, et al; European Uremic Toxin Work Group. Normal and pathologic concentrations of uremic toxins. J Am Soc Nephrol. 2012;23: 1258–1270. 10.1681/ASN.2011121175 22626821 PMC3380651

[7] Neirynck N, Glorieux G, Schepers E, Pletinck A, Dhondt A, Vanholder R. Review of protein-bound toxins, possibility for blood purification therapy. Blood Purif. 2013;35 Suppl 1: 45–50. 10.1159/000346223 23466378

[8] Meyer TW, Hostetter TH. Uremic solutes from colon microbes. Kidney Int. 2012;81: 949–954. 10.1038/ki.2011.504 22318422

[9] Ito S, Yoshida M. Protein-bound uremic toxins: new culprits of cardiovascular events in chronic kidney disease patients. Toxins (Basel). 2014; 6: 665–678. 24561478 10.3390/toxins6020665 PMC3942758

[10] Viaene L, Annaert P, de Loor H, Poesen R, Evenepoel P, Meijers B. Albumin is the main plasma binding protein for indoxyl sulfate and p-cresyl sulfate. Biopharm Drug Dispos. 2013;34: 165–175. 10.1002/bdd.1834 23300093

[11] Liabeuf S, Barreto DV, Barreto FC, Meert N, Glorieux G, Schepers E, et al; European Uraemic Toxin Work Group (EUTox). Free p-cresylsulphate is a predictor of mortality in patients at different stages of chronic kidney disease. Nephrol Dial Transplant. 2010;25: 1183–1191. 10.1093/ndt/gfp592 19914995

[12] Barreto FC, Barreto DV, Liabeuf S, Meert N, Glorieux G, Temmar M, et al; European Uremic Toxin Work Group (EUTox) Serum indoxyl sulfate is associated with vascular disease and mortality in chronic kidney disease patients. Clin J Am Soc Nephrol. 2009;4: 1551–1158. 10.2215/CJN.03980609 19696217 PMC2758258

[13] Lin CJ, Chen HH, Pan CF, Chuang CK, Wang TJ, Sun FJ, et al p-Cresylsulfate and indoxyl sulfate level at different stages of chronic kidney disease. J Clin Lab Anal. 2011;25: 191–197. 10.1002/jcla.20456 21567467 PMC6647585

[14] Huang ST, Shu KH, Cheng CH, Wu MJ, Yu TM, Chuang YW, et al Serum total p-cresol and indoxyl sulfate correlated with stage of chronic kidney disease in renal transplant recipients. Transplant Proc. 2012; 44: 621–624. 10.1016/j.transproceed.2011.11.023 22483453

[15] Melamed ML, Plantinga L, Shafi T, Parekh R, Meyer TW, Hostetter TH, et al Retained organic solutes, patient characteristics and all-cause and cardiovascular mortality in hemodialysis: results from the retained organic solutes and clinical outcomes (ROSCO) investigators. BMC Nephrol. 2013;14: 134 10.1186/1471-2369-14-134 23806101 PMC3698023

[16] Wu IW, Hsu KH, Hsu HJ, Lee CC, Sun CY, Tsai CJ, et al Serum free p-cresyl sulfate levels predict cardiovascular and all-cause mortality in elderly hemodialysis patients—a prospective cohort study. Nephrol Dial Transplant. 2012; 27: 1169–1175. 10.1093/ndt/gfr453 21891772

[17] Lin CJ, Wu CJ, Pan CF, Chen YC, Sun FJ, Chen HH. Serum protein-bound uraemic toxins and clinical outcomes in haemodialysis patients. Nephrol Dial Transplant. 2010;25: 3693–3700. 10.1093/ndt/gfq251 20466687

[18] Bammens B, Evenepoel P, Keuleers H, Verbeke K, Vanrenterghem Y. Free serum concentrations of the protein-bound retention solute p-cresol predict mortality in hemodialysis patients. Kidney Int. 2006;69: 1081–1087. 16421516 10.1038/sj.ki.5000115

[19] Dai WM, Yang B, Chu XY, Wang YQ, Zhao M, Chen L, et al Association between folate intake, serum folate levels and the risk of lung cancer: a systematic review and meta-analysis. Chin Med J (Engl). 2013; 126: 1957–1964. 23673118

[20] Lau J, Ioannidis JP, Schmid CH. Quantitative synthesis in systematic reviews. Ann Intern Med. 1997; 127: 820–826. 9382404 10.7326/0003-4819-127-9-199711010-00008

[21] Higgins JP, Thompson SG. Quantifying heterogeneity in a meta-analysis. Stat Med. 2002);21: 1539–1558. 12111919 10.1002/sim.1186

[22] DerSimonian R, Laird N. Meta-analysis in clinical trials. Control Clin Trials. 1986;7: 177–188. 3802833 10.1016/0197-2456(86)90046-2

[23] Egger M, Davey Smith G, Schneider M, Minder C. Bias in meta-analysis detected by a simple, graphical test. BMJ. 1997;315: 629–634. 9310563 10.1136/bmj.315.7109.629 PMC2127453

[24] Duval S, Tweedie R. Trim and fill: A simple funnel-plot-based method of testing and adjusting for publication bias in meta-analysis. Biometrics. 2000; 56:455–463. 10877304 10.1111/j.0006-341x.2000.00455.x

[25] Sutton AJ, Duval SJ, Tweedie RL, Abrams KR, Jones DR. Empirical assessment of effect of publication bias on meta-analyses. BMJ. 2000;320: 1574–1577. 10845965 10.1136/bmj.320.7249.1574 PMC27401

[26] Sutton AJ, Song F, Gilbody SM, Abrams KR. Modelling publication bias in meta-analysis: a review. Stat Methods Med Res. 2000;9: 421–445. 11191259 10.1177/096228020000900503

[27] Lin CJ, Pan CF, Chuang CK, Liu HL, Sun FJ, Wang TJ, et al Gastrointestinal-related uremic toxins in peritoneal dialysis: a pilot study with a 5-year follow-up. Arch Med Res. 2013;44: 535–541. 10.1016/j.arcmed.2013.09.007 24055267

[28] Lin CJ, Chuang CK, Jayakumar T, Liu HL, Pan CF, Wang TJ, et al Serum p-cresyl sulfate predicts cardiovascular disease and mortality in elderly hemodialysis patients. Arch Med Sci. 2013; 9: 662–668. 10.5114/aoms.2013.36901 24049526 PMC3776179

[29] Lin CJ, Liu HL, Pan CF, Chuang CK, Jayakumar T, Wang TJ, et al Indoxyl sulfate predicts cardiovascular disease and renal function deterioration in advanced chronic kidney disease. Arch Med Res. 2012; 43: 451–456. 10.1016/j.arcmed.2012.08.002 22885091

[30] Chen TC, Wang CY, Hsu CY, Wu CH, Kuo CC, Wang KC, et al Free p-cresol sulfate is associated with survival and function of vascular access in chronic hemodialysis patients. Kidney Blood Press Res. 2012;35: 583–588. 10.1159/000339709 22922348

[31] Wu IW, Hsu KH, Lee CC, Sun CY, Hsu HJ, Tsai CJ, et al p-Cresyl sulphate and indoxyl sulphate predict progression of chronic kidney disease. Nephrol Dial Transplant. 2011;26: 938–947. 10.1093/ndt/gfq580 20884620 PMC3042976

[32] Itoh Y, Ezawa A, Kikuchi K, Tsuruta Y, Niwa T. Protein-bound uremic toxins in hemodialysis patients measured by liquid chromatography/tandem mass spectrometry and their effects on endothelial ROS production. Anal Bioanal Chem. 2012;403: 1841–1850. 10.1007/s00216-012-5929-3 22447217

[33] Meijers BK, Claes K, Bammens B, de Loor H, Viaene L, Verbeke K, et al p-Cresol and cardiovascular risk in mild-to-moderate kidney disease. Clin J Am Soc Nephrol. 2010;5: 1182–1189. 10.2215/CJN.07971109 20430946 PMC2893077

[34] Rossi M, Campbell K, Johnson D, Stanton T, Pascoe E, Hawley C, et al Uraemic toxins and cardiovascular disease across the chronic kidney disease spectrum: an observational study. Nutr Metab Cardiovasc Dis. 2014;24: 1035–1042. 10.1016/j.numecd.2014.04.006 24880738

[35] Poesen R, Viaene L, Verbeke K, Augustijns P, Bammens B, Claes K, et al Cardiovascular disease relates to intestinal uptake of p-cresol in patients with chronic kidney disease. BMC Nephrol. 2014;15: 87 10.1186/1471-2369-15-87 24912660 PMC4064102

[36] Lin CJ, Pan CF, Chuang CK, Sun FJ, Wang DJ, Chen HH, et al P-cresyl sulfate is a valuable predictor of clinical outcomes in pre-ESRD patients. Biomed Res Int 2014;526932 10.1155/2014/526932 24592393 PMC3925630

[37] Vanholder R, Schepers E, Pletinck A, Nagler EV, Glorieux G. The uremic toxicity of indoxyl sulfate and p-cresyl sulfate: a systematic review. J Am Soc Nephrol. 2014; 25: 1897–1907. 10.1681/ASN.2013101062 24812165 PMC4147984

[38] Poveda J, Sanchez-Niño MD, Glorieux G, Sanz AB, Egido J, Vanholder R, et al p-Cresyl sulphate has pro-inflammatory and cytotoxic actions on human proximal tubular epithelial cells. Nephrol Dial Transplant. 2014;29: 56–64. 10.1093/ndt/gft367 24166466

[39] Rossi M, Campbell KL, Johnson DW, Stanton T, Vesey DA, Coombes JS, et al Protein-bound uremic toxins, inflammation and oxidative stress: a cross-sectional study in stage 3–4 chronic kidney disease. Arch Med Res. 2014; 45: 309–317. 10.1016/j.arcmed.2014.04.002 24751327

